# Granuphilin exclusively mediates functional granule docking to the plasma membrane

**DOI:** 10.1038/srep23909

**Published:** 2016-04-01

**Authors:** Kouichi Mizuno, Takuji Fujita, Hiroshi Gomi, Tetsuro Izumi

**Affiliations:** 1 Laboratory of Molecular Endocrinology and Metabolism, Department of Molecular Medicine, Institute for Molecular and Cellular Regulation, Gunma University, Gunma 371-8512, Japan; 2 Research Program for Signal Transduction, Division of Endocrinology, Metabolism and Signal Research, Gunma University Initiative for Advanced Research, Maebashi, Gunma 371-8512, Japan

## Abstract

In regulated exocytosis, it is generally assumed that vesicles must stably “dock” at the plasma membrane before they are primed to become fusion-competent. However, recent biophysical analyses in living cells that visualize fluorescent secretory granules have revealed that exocytic behaviors are not necessarily uniform: some granules beneath the plasma membrane are resistant to Ca^2+^ -triggered release, while others are accelerated to fuse without a pause for stable docking. These findings suggest that stable docking is unnecessary, and can even be inhibitory or nonfunctional, for fusion. Consistently, pancreatic β cells deficient in the Rab27 effector, granuphilin, lack insulin granules directly attached to the plasma membrane in electron micrographs but nevertheless exhibit augmented exocytosis. Here we directly compare the exocytic behaviors between granuphilin-positive and -negative insulin granules. Although granuphilin makes granules immobile and fusion-reluctant beneath the plasma membrane, those granuphilin-positive, docked granules release a portion of granuphilin upon fusion, and fuse at a frequency and time course similar to those of granuphilin-negative undocked granules. Furthermore, granuphilin forms a 180-nm cluster at the site of each docked granule, along with granuphilin-interacting Rab27a and Munc18-1 clusters. These findings indicate that granuphilin is an exclusive component of the functional and fusion-inhibitory docking machinery of secretory granules.

In regulated secretory cells, exocytosis takes place in response to an appropriate stimulus that typically increases free Ca^2+^ in the cytoplasm. In electron microscopy micrographs, a fraction of secretory vesicles are found attached to the target plasma membrane in a resting state. Such stably “docked” vesicles have generally been thought to be poised for release upon sensing secretagogues, and thus to fuse readily and promptly. Because the number of docked vesicles typically exceeds that of vesicles released by a brief stimulus (a readily releasable pool) in neuroendocrine cells^1^,^2^, it is assumed that a subset of docked vesicles are subsequently “primed” to acquire fusion competence. Thus, the current prevailing model postulates that all secretory vesicles follow the linear docking-priming-fusion pathway^3^,^4^.

However, direct observations of fluorescence-labeled secretory granules in living cells by total internal reflection fluorescence microscopy (TIRFM) have revealed that exocytic profiles are not necessarily uniform: for example in the case of pancreatic β cells, granules located both close to and relatively remote from the plasma membrane can fuse in parallel even during the first 1 minute after stimulation^5^,^6^,^7^. Furthermore, a significant fraction of fusing granules recruited from a distant cytoplasmic area only appear in the evanescent field within less than 50–100 ms of the fusion event, and thus, do not seem to pause to become stably docked. Those rapidly processed, fusing granules are also found in chromaffin cells^8^, and this kind of fusion without stable docking has been referred to as “crash fusion^4^”. Moreover, two-photon excitation of polar extracellular tracers in pancreatic acinar cells has shown that granules deep in the cell readily fuse to Ω-shaped membrane profiles of previously fused granules^9^. Such “sequential exocytosis” also indicates that granules possess fusion readiness without stable docking to the plasma membrane.

As to the molecular machinery, we previously showed that granuphilin (also known as Slp4), which is targeted to granules through an interaction with the small G-protein Rab27a^10^,^11^, is essential for granule docking, because granuphilin-null pancreatic β cells lack granules directly attached to the plasma membrane, as viewed under electron microscopy^7^,^12^. Surprisingly, despite this docking defect, those cells exhibit enhanced granule exocytosis in both resting and stimulated states. Reciprocally, overexpression of granuphilin accumulates granules close to the plasma membrane^13^ and inhibits their fusion^14^,^15^. The fusion-inhibitory effect of granuphilin has been proposed to reflect a specific interaction with the fusion-incompetent closed form of syntaxins^15^,^16^, members of soluble *N*-ethylmaleimide-sensitive factor attachment protein receptor (SNARE) proteins that mediate fusion reaction^17^. These findings indicate that granuphilin is essential for docking and defines a pathway that simultaneously imposes a fusion constraint on granules, and furthermore, that fusing granules can be recruited from a non-docked pool.

All these findings indicate that, contrary to the linear model, docking represents a temporarily fusion-reluctant state that prevents spontaneous fusion of incoming vesicles in the absence of an appropriate stimulus^7^,^18^,^19^. However, other researchers have suggested that this inhibitory docking represents dead-end docking, as distinct from normal functional docking^4^, although it is questionable whether such parallel docking pathways exist because granuphilin-null β cells almost completely lose stably docked granules^12^. To elucidate the functional significance of stable docking in regulated exocytosis, we visualized the exocytic behaviors of granuphilin-positive granules under TIRFM and the nanoscale distribution of granuphilin under direct stochastic optical reconstruction microscopy (dSTORM). Our results indicate that granuphilin is exclusively responsible for all functional docking, which makes granules temporarily fusion-inhibitory, yet still fusible given the appropriate stimulation.

## Results

### Granuphilin increases the density of granules beneath the plasma membrane

**Figure 1:**
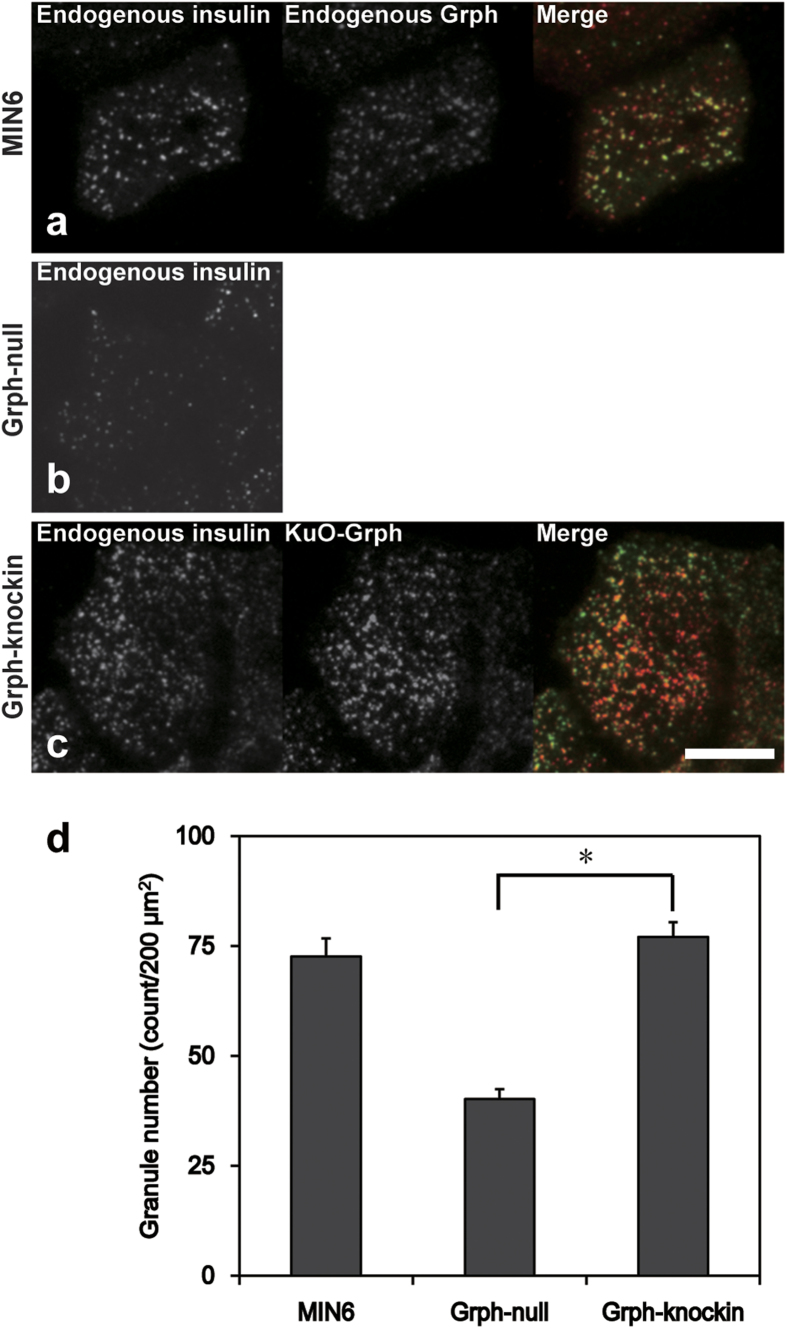
Granuphilin increases the density of granules beneath the plasma membrane. MIN6 (**a**), granuphilin (Grph)-null β cells (**b**) and Grph-null cells with exogenous KuO-Grph expression (Grph-knockin, (**c**)) were immunostained with anti-insulin antibody (green). MIN6 cells were further immunostained with anti-granuphilin antibody (red). Those cells were subjected to imaging by TIRFM, and the densities of granules were measured (**d**). Bar, 10 μm. *P < 0.0005.

MIN6 cells^20^, a widely used surrogate for pancreatic β cells, were coimmunostained with anti-insulin and anti-granuphilin antibodies, and were observed by TIRFM that specifically illuminates fluorescence in the area above the glass coverslip. Although TIRFM can capture insulin granules beneath the plasma membrane (72.6 ± 4.1 count/200 μm^2^; *n* = 8; Fig. 1a,d), it would not distinguish molecularly docked granules from those simply located near the plasma membrane. In fact, 78.0 ± 2.6% of insulin granules were granuphilin-positive, whereas the remaining 22.0 ± 2.6% were granuphilin-negative. Therefore, although most of the granules beneath the plasma membrane harbor granuphilin, a considerable number of them lack it. To specifically monitor the exocytic behaviors of granuphilin-positive granules in living cells, we labeled granules with fluorescent granuphilin in MIN6 cells. However, when we expressed exogenous granuphilin in the presence of endogenous granuphilin, overexpressed granuphilin markedly inhibited granule exocytosis, as reported previously^14^,^15^. Furthermore, rigorous comparison between granuphilin-positive and -negative granules is impossible in those cells because the contribution of unlabeled endogenous granuphilin cannot be assessed. Therefore, we newly generated β-cell lines lacking endogenous granuphilin by crossing granuphilin knockout mice^12^ with transgenic mice expressing the simian virus 40 (SV40) large T antigen gene under the insulin gene promoter^21^, similar to the method by which wild-type β-cell lines, including MIN6 cells, have been established^20^,^22^,^23^. In these cells, the density of insulin granules under TIRFM was 40.2 ± 2.3 count/200 μm^2^ (*n* = 8), much lower than that in MIN6 cells (Fig. 1b,d). However, when they were infected with an adenovirus encoding Kusabira-Orange 1-granuphilin (KuO-Grph), the density of granules increased to 77.0 ± 3.4 count/200 μm^2^ (*n* = 8), similar to that in MIN6 cells (Fig. 1c,d). We labeled granules with fluorescent granuphilin to a similar extent as that done for endogenous granuphilin in wild-type cells. Namely, 80.4 ± 2.3% of insulin granules were granuphilin-positive and the remaining 19.6 ± 2.3% were granuphilin-negative under TIRFM, which values were close to those found in MIN6 cells. These findings indicate that granuphilin has an activity to tether granules stably to the plasma membrane, as suggested previously^13^.

### Granuphilin immobilizes granules beneath the plasma membrane

**Figure 2:**
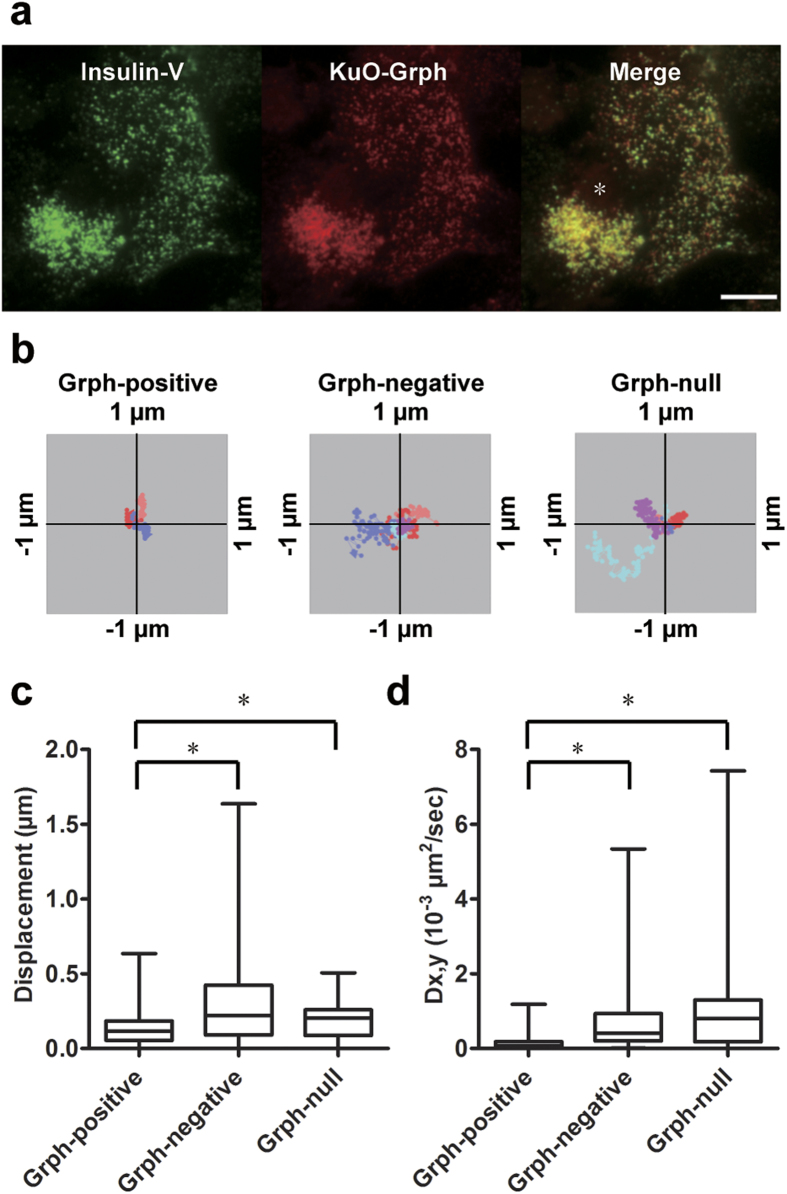
Granuphilin-positive granules are immobile. Granuphilin (Grph)-null β cells expressing Insulin-V and KuO-Grph were incubated in low glucose (2.8 mM) Krebs Ringer buffer and were observed by TIRFM. Although some cells ((**a**) asterisk) overexpressed KuO-Grph and abnormally accumulated insulin granules beneath the plasma membrane, those cells were excluded from subsequent analyses. Bar, 10 μm. Representative tracks of Grph-positive (*n* = 3) and -negative insulin granules (*n* = 5) in Grph-knockin cells, and those of granules (*n* = 5) in Grph-null cells are shown as centroid plots (**b**). The maximum displacement (**c**) and 2D diffusion coefficient (Dx,y; (**d**)) were calculated from tracks of Grph-positive (*n* = 210 for (**c**) and 238 for (**d**)) and -negative granules (64 for (**c**) and 63 for (**d**)) from 8 Grph-knockin cells, and granules from 4 Grph-null cells (*n* = 43 for (**c**) and (**d**)). A box and a bar within the box indicate the 25–75% range and a median value, respectively, whereas outer bars represent minimum and maximum values. The significance of differences was assessed by a Mann-Whitney U test. *P < 0.0005.

We then infected the granuphilin-null β cells with adenoviruses encoding preproinsulin-Venus (Insulin-V) and KuO-Grph. In these cells, 85.4 ± 3.7% (73.8 ± 6.6 count/200 μm^2^; *n* = 15) of visible insulin granules were granuphilin-positive and 14.6 ± 3.7% (12.2 ± 2.8 count/200 μm^2^) were granuphilin-negative under TIRFM (Fig. 2a). The slight increase in the density of visible granules compared with that obtained from endogenous insulin immunostaining (Fig. 1c,d) may be due to difference in methods for granule labeling. Granuphilin-positive granules exhibited severely restricted mobility in a resting state. The median values of the maximum displacement and a two-dimensional diffusion coefficient Dx,y for granuphilin-positive granules were significantly lower than those for granuphilin-negative granules, although Dx,y describes the motion assuming unrestricted diffusion (Fig. 2b–d and Supplementary Video S1). By contrast, in the same cells without exogenous KuO-Grph expression, the mobility of granules beneath the plasma membrane was not restricted (Fig. 2b–d and Supplementary Video S2). These findings indicate that granuphilin molecularly tethers granules to the plasma membrane and makes them immobile in living cells, and furthermore, suggest that granules visible under TIRFM are not necessarily directly attached or stably tethered to the plasma membrane.

### Molecularly docked granules are fusion-reluctant

**Figure 3:**
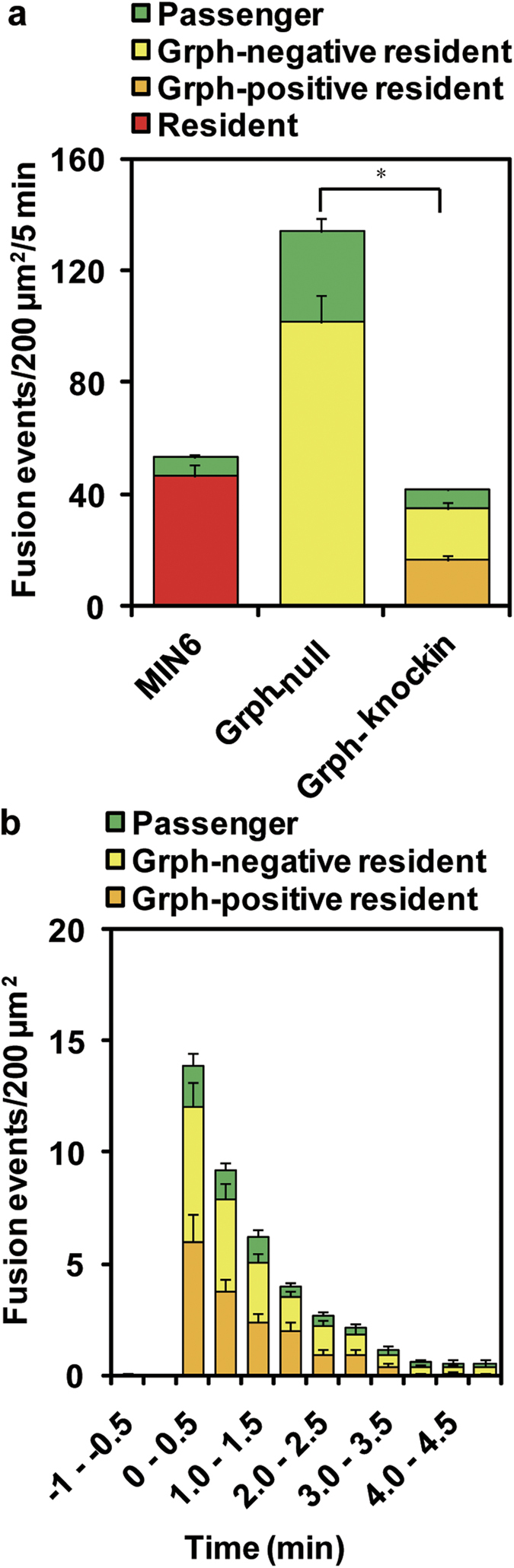
Granuphilin-positive granules are fusion-reluctant. (**a**) The histogram shows the number of fusion events per 200 μm^2^ during a 5-min stimulation with 60 mM KCl in MIN6 cells (*n* = 15), granuphilin (Grph)-null β cells (*n* = 16), and Grph-null β cells expressing KuO-Grph (Grph-knockin; *n* = 16). Fusion events were categorized into “*resident*” (discriminating between Grph-positive and -negative granules, when applicable) or “*passenger*” (see Methods). In MIN6 cells, all *residents* were summed up into one category without Grph-positive and -negative classification, because KuO-Grph was not expressed. In Grph-null cells without KuO-Grph expression, all *residents* were considered Grph-negative, because of the absence of granuphilin. *P < 0.0001. (**b**) The number of fusion events from each type of exocytosis is plotted per 0.5-min interval in Grph-knockin cells.

We next compared depolarization-induced fusion rates between granuphilin-positive and -negative granules under TIRFM. Although the fusion rates in granuphilin-null β cells were more than 2-fold higher than those in MIN6 cells, presumably due to the absence of granuphilin, they decreased to a comparable level after the expression of KuO-Grph (Fig. 3a). As was previously found in MIN6 and mouse pancreatic β cells^7^,^24^, most of the depolarization-induced fusion (84.2%) was derived from the granules called *residents* that had remained in an evanescent field for more than an interval of one frame (103 ms) before fusion, whereas the rest (15.8%) involved granules called *passengers* that were newly recruited from outside of the evanescent field and immediately fused within 103 ms in those KuO-Grph knockin β cells (Fig. 3a and Supplementary Video S3). In *passengers* that are visible only in one frame, by definition (see Methods), it was not possible to judge whether they had associated with granuphilin before fusion, although no granuphilin fluorescence was seen on *passengers* at the time of fusion (see an example in Supplementary Video S4), and granuphilin, if present, is unlikely to tether those relatively distant granules to the plasma membrane. We thus focused on *resident* granules for which we could definitely judge the presence or absence of granuphilin prior to fusion. Within the 84.2% of fusion probability of *residents*, granuphilin-positive and -negative granules showed similar fusion numbers (40.1% vs. 44.1%; Fig. 3a) and also fused in parallel, even at early time points after stimulation (Fig. 3b). Considering that 85.4% of *resident* granules were granuphilin-positive and 14.6% were granuphilin-negative before stimulation (Fig. 2a), the fusion probability of granuphilin-positive *residents* was less than 20% of that of granuphilin-negative *residents*.

### Granuphilin dissociates from docked granules upon fusion

**Figure 4:**
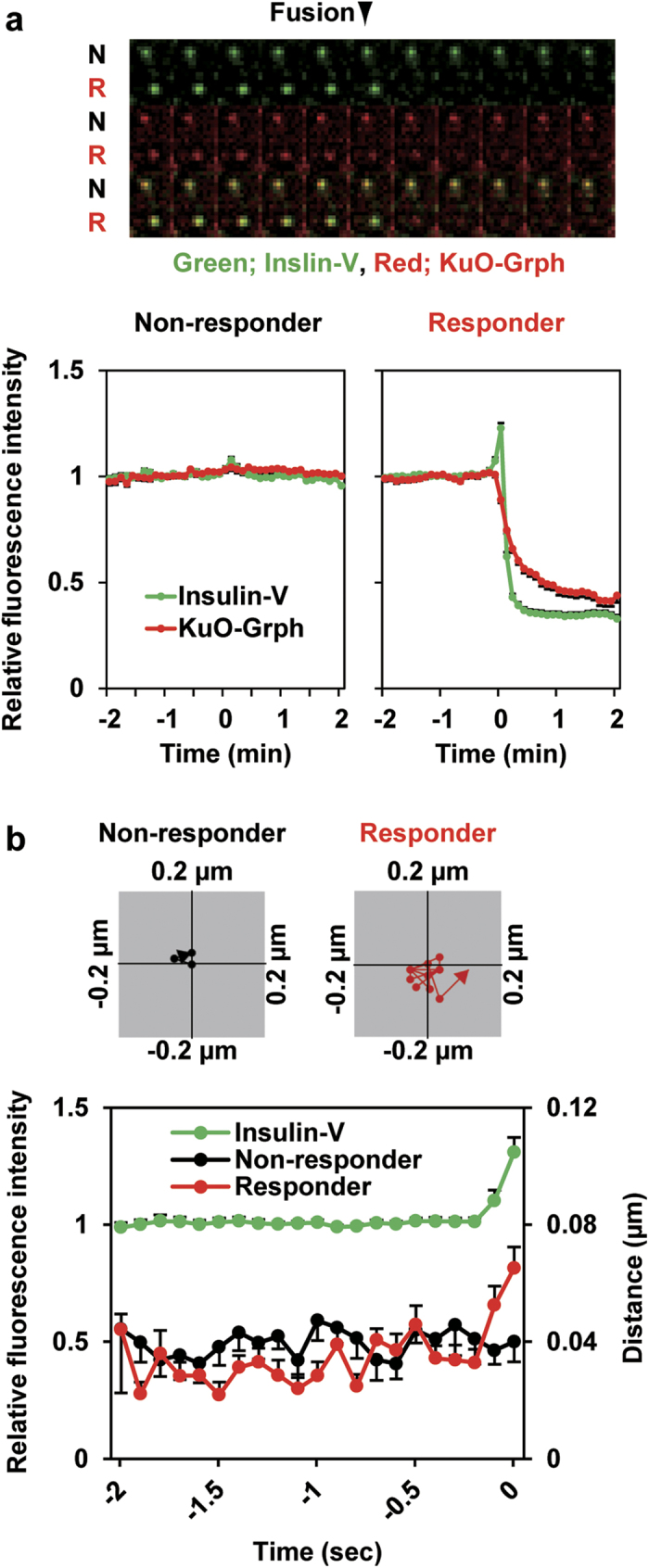
Docked granules release granuphilin and become mobile upon fusion. The fluorescence profiles of insulin and granuphilin upon fusion (time zero) were monitored for granuphilin-positive *residents* in Grph-knockin cells. (**a**) Kymographs (top) show the fluorescence profiles of Insulin-V (green) and KuO-Grph (red) of fused granules (*responder*, R) and neighboring inert granules (*non-responder*, N). Bottom panels show normalized changes in fluorescence intensities of *non-responders* (*n* = 44 from 12 cells) and *responders* (*n* = 147 from 14 cells). (**b**) Upper panels show centroid plots of a representative granule track from a *non-responder* and a *responder*. Bottom panel plots distance of fluorescence center points of individual *responders* (red, *n* = 36) and *non-responders* (black, *n* = 20) between time points, as well as relative fluorescence intensity of Insulin-V in *responders* (green, *n* = 40), from 5 cells.

We then monitored granule-associated granuphilin fluorescence, because fusion-inhibitory granuphilin could be removed from fusing granules. We found that the intensity of granuphilin fluorescence significantly declined upon fusion, when the intensity of insulin fluorescence transiently increased by neutralization of acidic pH in granules, and subsequently decreased due to extracellular diffusion (Fig. 4a and Supplementary Video S5). The decreases in granuphilin fluorescence on fused granules were 11.0 ± 1.4% at time zero, 45.1 ± 2.1% at 0.5 min, and 53.6 ± 2.5% at 1 min, after fusion (*n* = 147 from 14 cells; Fig. 4a, lower right panel). Furthermore, the motility of granuphilin-positive granules increased just before fusion (Fig. 4b and Supplementary Video S5), consistent with the previous finding in chromaffin cells^25^. It is currently unknown how much portion of granule-associated granuphilin is actually involved in docking by interacting with syntaxin and/or phospholipids on the plasma membrane. A fractional dissociation of granuphilin might make docked granules mobile and fusible.

### Granuphilin forms a 180-nm cluster on the granule beneath the plasma membrane

**Figure 5:**
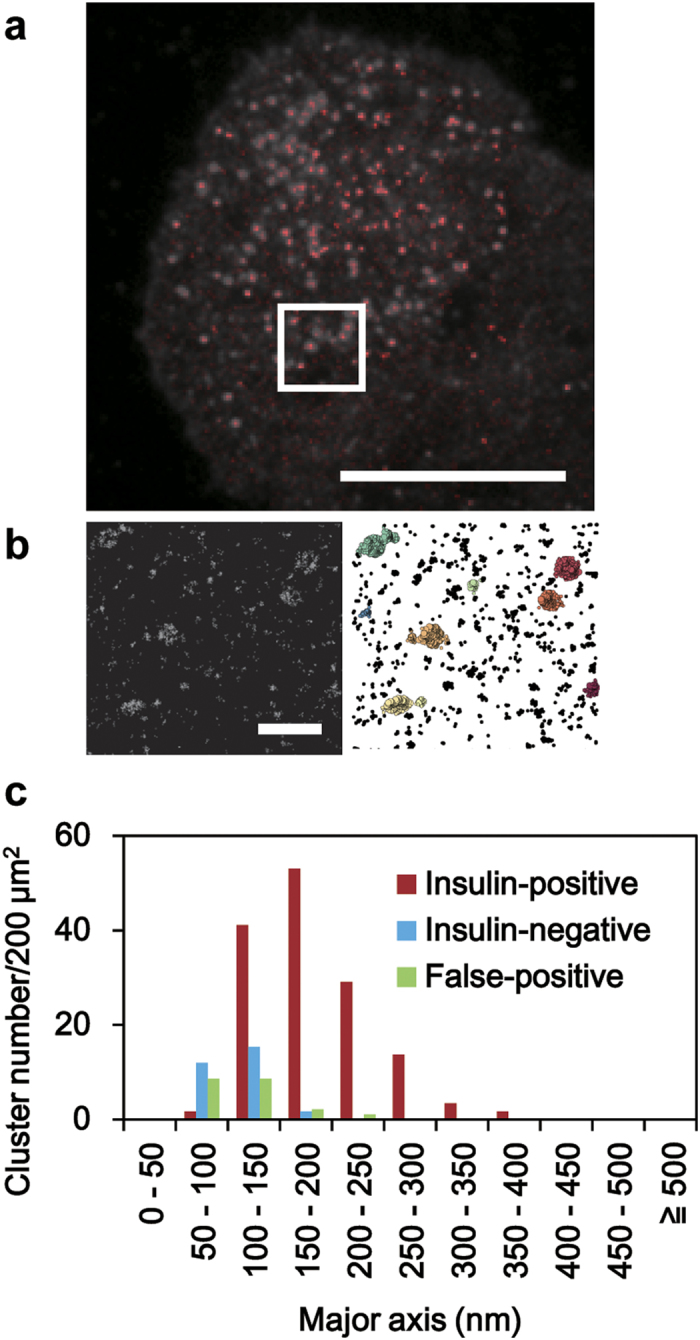
Granuphilin forms a 180-nm cluster on each docked granule. MIN6 cells were immunostained with anti-granuphilin and anti-insulin antibodies. (**a**) A merged image of endogenous insulin (gray) by TIRFM and endogenous granuphilin (red) by dSTORM. Bar, 5 μm. (**b**) A dSTORM image of granuphilin ((**b**), left) and its clusters identified by DBSCAN ((**b**), right) from a boxed region in (**a)**. Bar, 500 nm. (**c**) Histograms show the distribution of the major axis of granuphilin clusters. Granuphilin clusters identified in MIN6 cells are divided into two classes based on their location, either on (red) or not on (blue) insulin granules. The distribution of false-positive granuphilin clusters identified in corresponding areas of granuphilin-null β cells (green) is also shown.

To determine the distribution of granuphilin beneath the plasma membrane at the nanoscale, MIN6 cells were immunostained with anti-granuphilin and Alexa 647-labeled secondary antibodies and were observed by dSTORM. The same MIN6 cells were simultaneously immunostained with anti-insulin antibody and observed by TIRFM to distinguish the granuphilin clusters on insulin granules from those not on the granules. To confirm the specificity of our anti-peptide antibody against granuphilin, granuphilin-null β cells were also immunostained and observed by both microscopies under the same conditions. To identify granuphilin clusters, dSTORM images were analyzed using a clustering algorithm, density-based spatial clustering of applications with noise (DBSCAN)^26^, which is robust to solitary noise and has been used for many super-resolution microscopic analyses^27^,^28^,^29^. We then draw an ellipse onto a scatter plot that contains 95% of the granuphilin location points to estimate the cluster sizes and shapes. Granuphilin formed a cluster specifically at the positions of granules underneath the plasma membrane in MIN6 cells (Fig. 5a,b), consistent with its role in granule docking. The density and major axis of such insulin-positive clusters were 0.38 clusters/μm^2^ and 185 ± 6 nm, respectively (Table 1 and Fig. 5c). By contrast, the density of insulin-negative granuphilin clusters was much lower, 0.08 clusters/μm^2^, and their major axis was much smaller, 107 ± 6 nm. Most of those small clusters were considered to be derived from non-specific immunostaining, because similar numbers (0.10 clusters/μm^2^) and sizes (112 ± 11 nm) of clusters were found in granuphilin-null β cells (Table 1 and Fig. 5c). Furthermore, the mean localizations of granuphilin molecules that made up a cluster on each insulin granule were 173 ± 10, whereas the corresponding localizations in a cluster not associated with granules in MIN6 cells and that found in granuphilin-null cells were much lower, 47 ± 4 and 38 ± 5, respectively (Table 1). Because Alexa fluorophores recover to a fluorescent state from a dark state multiple times before photobleaching in buffer containing an oxygen scavenging system and a thiol reagent under continuous illumination^30^, a single fluorophore can be detected several times during image acquisition. Therefore, the detected number of protein localizations that make up a cluster does not necessarily indicate the actual number of molecules. Nonetheless, our findings indicate that most of the small clusters (<100 nm in major axis, <100 localizations) are false-positive or, even if true-positive, are nonfunctional to dock granules. Thus, super-resolution imaging revealed that granuphilin molecules are specifically concentrated at the site of every docked granule.

**Figure 6:**
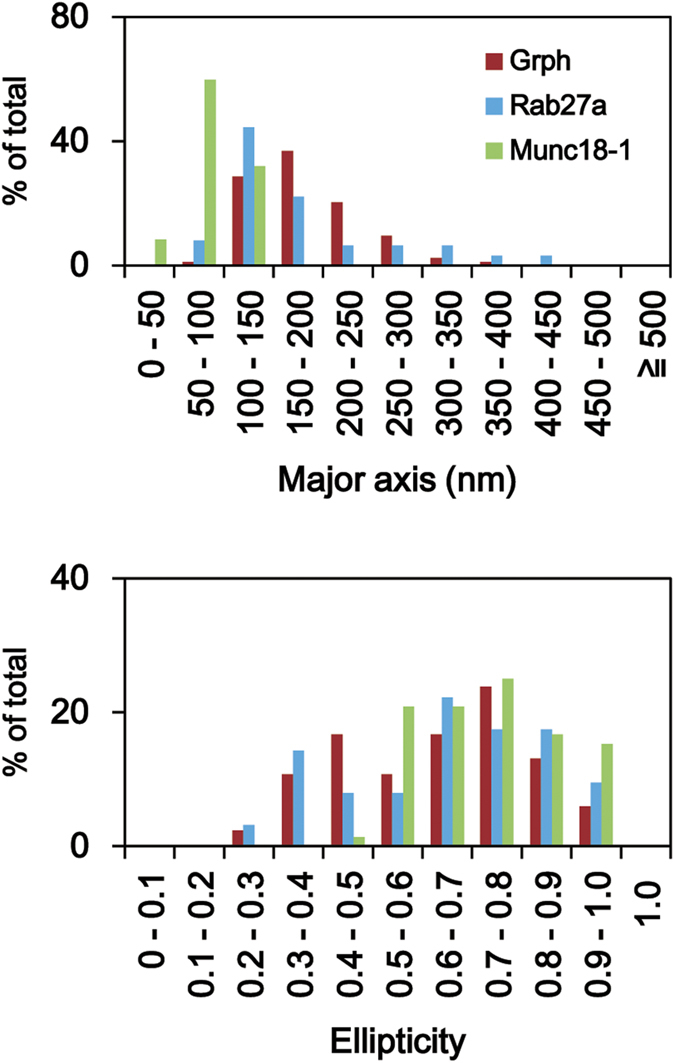
Comparison of cluster parameters among granuphilin, Rab27a, and Munc18-1. MIN6 cells were co-immunostained with anti-granuphilin antibody and either anti-Rab27a or anti-Munc18-1 antibody, and were imaged as in Fig. 5. The Rab27a and Munc18-1 clusters associated with granuphilin were further analyzed. Histograms show length of major axis (upper) and ellipticity (ratio of major and minor axes representing cluster shape; lower) of granuphilin-positive Rab27a (blue) and Munc18-1 (green), along with insulin-positive granuphilin (from Fig. 5, red).

Because granuphilin is thought to mediate granule docking by linking Rab27a on the granule membrane and the syntaxin-1a-Munc18-1 complex on the plasma membrane^31^, we next investigated the localization of Rab27a and Munc18-1 in MIN6 cells using dSTORM. To focus on the molecules involved in granule docking, we co-immunostained MIN6 cells with anti-granuphilin antibody and analyzed the Rab27a and Munc18-1 clusters that associated with the granuphilin clusters (Supplementary Fig. S1), because Rab27a may also bind other Rab27 effectors expressed in β cells^32^,^33^,^34^,^35^, and because Munc18-1 forms a complex with syntaxin-1a on the plasma membrane independent of docked granules^27^. The protein localizations, sizes, and shapes of granuphilin-positive Rab27a clusters were remarkably similar to those of insulin-positive granuphilin clusters (Table 1 and Fig. 6). By contrast, granuphilin-positive Munc18-1 clusters were made up of a higher number of protein localizations, were smaller in size, and relatively rounded, compared with the granuphilin and Rab27a clusters (Table 1 and Fig. 6).

## Discussion

In the present study, we closely examined assumptions within the accepted view about the functional meaning of docking in regulated exocytosis by simultaneously visualizing both granule cargo, insulin, and docking machinery, granuphilin, in living cells. First, we showed that granuphilin increases the density of granules beneath the plasma membrane and decreases their mobility in a resting state. Furthermore, granuphilin remains with the granules until they become mobile and fuse after stimulation. These findings indicate that granuphilin is not only essential for stable docking of secretory granules^12^ but also is a direct component of the docking machinery. This conclusion is in line with the general role of Rab and its effector proteins in tethering vesicles to the target membrane^36^, but is incompatible with the predominant hypothesis that docking involves the pairing of SNARE proteins between the opposing membranes because granuphilin preferentially interacts with the closed form of syntaxin that prevents the SNARE assembly^15^. Very recently, a work using two-photon fluorescence lifetime imaging shows that *trans*-SNARE complexes are not formed in a resting state of β cells, and assembled only shortly prior to insulin exocytosis, although they accumulate in the active zone in presynaptic boutons^37^. This finding also suggests that SNARE proteins are not preassembled on existing, stably docked granules.

Second, we demonstrated the much lower fusion probability of granuphilin-positive docked granules compared with granuphilin-negative undocked granules, although docking has generally been thought to be a requisite step preceding fusion^4^. At the same time, we showed in living cells that a substantial fraction of granuphilin-positive granules do fuse after stimulation with similar frequency and time course as granuphilin-negative granules do, although fusion-inhibitory granuphilin has been suggested to cause dead-end docking apart from functional docking^4^. Therefore, granuphilin-mediated docking is neither a prerequisite for fusion nor an off-pathway leading to a dead end. The syntaxin-1a-Munc18-1 complex formation as a platform of granuphilin on the plasma membrane is considered to represent a physiologically relevant pathway protecting nearby granules from spontaneous fusion in regulated exocytosis^7^,^18^,^19^.

Finally, we investigated the nanoscale organization of the granule docking machinery. To our knowledge, this is the first study to investigate how molecules connecting two apposed membranes are distributed at the nanoscale. We showed that granuphilin molecules form a cluster on each docked granule, consistent with the phenotype of granuphilin-null β cells^12^. This finding is compatible with the concept that granuphilin is responsible for all stable granule docking, but is inconsistent with the notion that granuphilin is only involved in a particular type of stable docking, such as dead-end docking. The size of the granuphilin cluster (180 nm) is about half that of the mouse insulin granule (350 nm) estimated from electron micrographs^12^, which suggests that the granules are docked to a plasma membrane having the sort of restricted surface area (~10%) characteristic of a sphere. We also found that granuphilin-interacting Rab27a and Munc18-1 form clusters on granuphilin-positive docked granule areas. It has been shown by super-resolution microscopy that the t-SNARE proteins, syntaxin-1a/b and SNAP-25, form 50- to 100-nm clusters on the plasma membrane in PC12 cells^38^,^39^. Furthermore, those t-SNARE proteins and Munc18-1 are found in the same 50- to 100-nm clusters in primary mouse neuronal cells^27^, which is consistent with the size of Munc18-1 clusters (90 nm) found in MIN6 cells in the present study. Granuphilin interacts with Rab27a through its N-terminal domain^11^, with syntaxin-1a-Munc18-1 complex through its central linker domain^40^, and with plasma membrane phospholipids through its C-terminal C2 domains^10^. Thus, peripheral N-terminal interaction with a spherical granule membrane and/or C-terminal interaction with the plasma membrane may cause granuphilin clusters to be larger than the centrally located syntaxin-1a-Munc18-1 clusters, although we did not investigate the distribution of syntaxin-1a due to the unavailability of a specific antibody compatible with whole-cell immunostaining.

## Methods

### Establishment of pancreatic β-cell lines deficient in granuphilin

All animal experiment protocols were performed according to the guidelines of the Animal Care and Experimentation Committee, Gunma University, and were approved by the Committee (the approved number: 14-018). Granuphilin knockout mice^12^ were crossed with heterozygous transgenic mice that target expression of the SV40 large T antigen gene under the insulin gene promoter^21^. Granuphilin-null β-cell lines were established from pancreatic islets obtained from 8- to 10-week-old mice that were homozygous for the granuphilin-null allele and heterozygous for the SV40 large antigen gene. The isolated islets were cultured in 35-mm dishes with RPMI medium supplemented with 10% fetal calf serum (FCS) for 1–2 days. They were then transferred into gelatin-coated 24-well dishes per single islet with high glucose (25 mM) Dulbecco’s modified Eagle’s medium supplemented with 15% FCS and 6 mM L-glutamine. After cell proliferation, each islet was digested with trypsin-EDTA and was transferred into a 48-well dish. Immortalized cells were expanded in 85-mm dishes and were frozen as cell stocks. These cells, as well as MIN6 cells, were maintained in the high glucose medium supplemented with 15% FCS and 55 mM 2-mercaptoethanol in a humidified incubator with 95% air and 5% CO_2_ at 37 °C.

### Construction of plasmids and recombinant adenoviruses

The construction of Insulin-V was previously described^34^. The construction of KuO-Grph was made by subcloning murine granuphilin cDNA into phmKO1-MC1 (MBL). For generation of the recombinant adenovirus, the cDNA fragment was ligated into a pAxCAwt cosmid vector (Takara Bio), which was then transfected into HEK293 cells with adenovirus genome DNA-TPC (Takara Bio). Granuphilin-null β cells or MIN6 cells were infected with adenoviruses at MOI 10 for 2 days.

### TIRFM

MIN6 and granuphilin-null β cells cultured on cover glasses were fixed with 3.7% formaldehyde and were immunostained as described previously^34^ with guinea-pig anti-porcine insulin serum (a gift from H. Kobayashi, Gunma University) at a 1:1000 dilution followed by Alexa 488-conjugated secondary antibody (Invitrogen) at a 1:500 dilution for 1 h at room temperature. MIN6 cells were coimmunostained with 10 μg/ml of anti-granuphilin antibody αGrp-N^11^ followed by Alexa 568-conjugated secondary antibody (Invitrogen).

Sequential multi-color TIRFM was performed on an inverted microscope Eclipse with an Apo TIRF 100 × /1.49 oil objective lens (Nikon). The penetration depth of the evanescent field calculated from the device parameters was 100 nm. Venus was excited using a 488-nm solid-state laser, whereas KuO was excited using a 561-nm laser. Excitation illumination was synchronously delivered from an acousto-optic tunable filter controlled laser launch with an electron multiplying charge-coupled device (EM-CCD) camera, the iXon DU-897 (Andor Technology), controlled by NIS-element software (Nikon). A quadra-band filter set or dual-band filter set (LF405/488/561/635-A, LF488/561-A; Semrock) was applied on a light path. Images were acquired every 103 ms and the image sequences were processed by a no neighbor deconvolution filter using Aquacosmos software (Hamamatsu Photonics). The positions of insulin granules in an x,y plane parallel to the membrane glass interface were determined by their intensity and size using a quantification module provided by Volocity software version 5.3 (PerkinElmer). The mass centers of contiguous bright pixels were tracked over the sequence. For each insulin granule trajectory, the mean square displacement.in the x,y plane was calculated from the straight line distance between the first and last centroids in a track of 100 time points. A two-dimension diffusion constant Dx,y was derived from the slope of the curve (slope = 4 × Dx,y). Granuphilin-positive and -negative granules were determined whether the granuphilin-fluorescent intensity in granule positions was more than twice that in neighboring non-granular regions.

A single-cell insulin release assay was performed as described previously^7^,^34^, with modifications. Briefly, granuphilin-null β cells were infected with an adenovirus encoding Insulin-V and KuO-Grph for visualization of granuphilin-positive insulin granules and were further cultured for 2 days. TIRFM was performed on a stage incubation system (Tokai Hit) maintained at 37 °C. Image sets from Insulin-V and KuO-Grph were acquired every 103 ms. After preincubation at 37 °C for 30 min in Krebs Ringer buffer containing 15 mM HEPES, pH 7.4, 120 mM NaCl, 5 mM KCl, 2 mM CaCl_2_, 1 mM MgCl_2_, 12 mM NaHCO_3_, 0.3% bovine serum albumin (BSA), and 2.8 mM glucose, the cells were stimulated for 5 min by addition of an equal volume of the buffer containing 10 mM NaCl and 115 mM KCl instead of 120 mM NaCl and 5 mM KCl. A fusion event with a flash was manually selected and assigned to one of three categories: “granuphilin-positive residents” that located in an evanescent field with granuphilin before fusion, “granuphilin-negative residents” that located in an evanescent field without granuphilin before fusion, and “passengers” that were newly recruited from outside of an evanescent field and fused to the plasma membrane immediately within an interval of one frame (103 ms). The average fluorescence intensity of individual vesicles was calculated in a 0.8 μm × 0.8 μm square placed over the granule center.

### dSTORM

Cells cultured in Lab-Tek II chambered cover glass (Nunc) were fixed with 3% paraformaldehyde and 0.1% glutaraldehyde in phosphate-buffered saline (PBS) for 30 min at room temperature. After rinsing three times with PBS, the cells were treated with freshly prepared reducing buffer containing 0.1% NaBH_4_ in PBS for 10 min, and were permeabilized and blocked with the blocking buffer containing 3% BSA and 0.2% Triton X-100 in PBS for 2 h. Then, the cells were incubated with anti-insulin serum or 10 μg/ml of αGrp-N, anti-Rab27a (BD Biosciences), or anti-Munc18-1 (BD Biosciences) antibodies at 4 °C overnight. After rinsing three times with washing buffer containing 0.2% BSA and 0.05% Triton X-100 in PBS, the cells were stained with Alexa 488- and 647-conjugated secondary antibodies at 4 °C for 1 h. The imaging buffer was freshly prepared before imaging by mixing buffer (50 mM Tris-HCl, pH 8.0, 10 mM NaCl, and 10% glucose), an oxygen scavenging system (1 M mercaptoethylamine in 0.25 M HCl), and GLOX solution (8 mM Tris-HCl, pH 8.0, 40 mM NaCl, 56 mg/ml glucose oxidase, and 3.4 mg/ml catalase) at a volume ratio of 620:70:7. The chambered cover glass filled with the imaging buffer was covered with a lid and sealed with parafilm.

dSTORM imaging was performed on an N-STORM system with an Apo TIRF 100/1.45 oil objective lens (Nikon). Two continuous wave laser beams (405 nm and 647 nm) were used simultaneously for activation and read-out for Alexa 647. The image was acquired in the presence of an enzymatic oxygen scavenging system and a millimolar concentration of thiol to induce blinking on a millisecond time scale. The laser powers were chosen to ensure that the fraction of activated fluorophores at any given time would be sufficiently low to enable recognition of individual fluorophores. Fluorescent light was spectrally filtered with a TIRF-Quad Filterset 405/488/561/647 (Chroma) and was imaged with an iXon3 897 Single Photon EMCCD camera (Andor Technology). Typically, we recorded 50,000 frames at rates of ~55 Hz. Applying a laser power of 200 milliwatts at 647 nm, we detected ~3,000 photons per molecule and frame, corresponding to a localization precision of less than ~20 nm. Localizations of fluorescent molecules were extracted by NIS-Elements software. All localizations in the dSTORM images (x,y coordinates of the center of mass of each fluorophore) were systematically scanned by a clustering algorithm, DBSCAN, which is packaged into scikit-learn 0.16.1, an open-source machine learning library for the Python3.4 programming language. DBSCAN requires two parameters: the eps, a distance from the core point, and the minPts, a minimum number of points required to form a cluster. Although any setting affects cluster detection, the validity of clusters was confirmed by visual inspection. In each cluster identified by DBSCAN, an ellipse containing 95% of the protein location points was drawn onto a scatter plot, and the length of a major axis and the ratio of major and minor axes (ellipticity) were obtained as elliptic parameters.

### Statistical analysis

All quantitative data are expressed as means ± SEM, unless otherwise indicated. The significance of differences was assessed by a Mann-Whitney U test, using GraphPad Prism software.

## Additional Information

**How to cite this article**: Mizuno, K. *et al.* Granuphilin exclusively mediates functional granule docking to the plasma membrane. *Sci. Rep.* **6**, 23909; doi: 10.1038/srep23909 (2016).

## Supplementary Material

Supplementary Information

Supplementary Video S1

Supplementary Video S2

Supplementary Video S3

Supplementary Video S4

Supplementary Video S5

## Figures and Tables

**Table 1:** Characteristics of granuphilin, Rab27a, and Munc18-1 clusters.

	Cluster number*	Localizations	Major axis (nm)	Ellipticity
Insulin-positive granuphilin	84	173.1 ± 9.9	184.7 ± 6.0	0.63 ± 0.02
Insulin-negative granuphilin	17	46.7 ± 4.0	107.0 ± 6.1	0.60 ± 0.04
False-positive granuphilin	19	37.8 ± 4.6	111.7 ± 10.7	0.57 ± 0.04
Granuphilin-positive Rab27a	63	198.1 ± 15.6	177.7 ± 10.6	0.65 ± 0.02
Granuphilin-positive Munc18-1	72	353.0 ± 24.9	85.8 ± 3.2	0.73 ± 0.02

^*^The number of clusters identified by DBSCAN in 23 areas (each area ~8 μm^2^) from 3 cells.

## References

[b1] Steyer J. A., Horstmann H. & Almers W. Transport, docking and exocytosis of single secretory granules in live chromaffin cells. Nature 388, 474–478 (1997). 9242406 10.1038/41329

[b2] Plattner H., Artalejo A. R. & Neher E. Ultrastructural organization of bovine chromaffin cell cortex-analysis by cryofixation and morphometry of aspects pertinent to exocytosis. J Cell Biol 139, 1709–1717 (1997). 9412466 10.1083/jcb.139.7.1709 PMC2132648

[b3] Rorsman P. & Renström E. Insulin granule dynamics in pancreatic beta cells. Diabetologia 46, 1029–1045 (2003). 12879249 10.1007/s00125-003-1153-1

[b4] Verhage M. & Sørensen J. B. Vesicle docking in regulated exocytosis. Traffic 9, 1414–1424 (2008). 18445120 10.1111/j.1600-0854.2008.00759.x

[b5] Ohara-Imaizumi M. *et al.* TIRF imaging of docking and fusion of single insulin granule motion in primary rat pancreatic beta-cells: different behaviour of granule motion between normal and Goto-Kakizaki diabetic rat beta-cells. Biochem J 381, 13–18 (2004). 15128287 10.1042/BJ20040434 PMC1133756

[b6] Shibasaki T. *et al.* Essential role of Epac2/Rap1 signaling in regulation of insulin granule dynamics by cAMP. Proc Natl Acad Sci USA 104, 19333–19338 (2007). 18040047 10.1073/pnas.0707054104 PMC2148290

[b7] Kasai K., Fujita T., Gomi H. & Izumi T. Docking is not a prerequisite but a temporal constraint for fusion of secretory granules. Traffic 9, 1191–1203 (2008). 18397364 10.1111/j.1600-0854.2008.00744.x

[b8] Allersma M. W., Wang L., Axelrod D. & Holz R. W. Visualization of regulated exocytosis with a granule-membrane probe using total internal reflection microscopy. Mol Biol Cell 15, 4658–4668 (2004). 15282339 10.1091/mbc.E04-02-0149 PMC519157

[b9] Nemoto T. *et al.* Sequential-replenishment mechanism of exocytosis in pancreatic acini. Nat Cell Biol 3, 253–258 (2001). 11231574 10.1038/35060042

[b10] Wang J., Takeuchi T., Yokota H. & Izumi T. Novel rabphilin-3-like protein associates with insulin-containing granules in pancreatic beta cells. J Biol Chem 274, 28542–28548 (1999). 10497219 10.1074/jbc.274.40.28542

[b11] Yi Z. *et al.* The Rab27a/granuphilin complex regulates the exocytosis of insulin-containing dense-core granules. Mol Cell Biol 22, 1858–1867 (2002). 11865063 10.1128/MCB.22.6.1858-1867.2002 PMC135591

[b12] Gomi H., Mizutani S., Kasai K., Itohara S. & Izumi T. Granuphilin molecularly docks insulin granules to the fusion machinery. J Cell Biol 171, 99–109 (2005). 16216924 10.1083/jcb.200505179 PMC2171228

[b13] Torii S., Takeuchi T., Nagamatsu S. & Izumi T. Rab27 effector granuphilin promotes the plasma membrane targeting of insulin granules via interaction with syntaxin 1a. J Biol Chem 279, 22532–22538 (2004). 15028737 10.1074/jbc.M400600200

[b14] Coppola T. *et al.* Pancreatic β-cell protein granuphilin binds Rab3 and Munc-18 and controls exocytosis. Mol Biol Cell 13, 1906–1915 (2002). 12058058 10.1091/mbc.02-02-0025 PMC117613

[b15] Torii S., Zhao S., Yi Z., Takeuchi T. & Izumi T. Granuphilin modulates the exocytosis of secretory granules through interaction with syntaxin 1a. Mol Cell Biol 22, 5518–5526 (2002). 12101244 10.1128/MCB.22.15.5518-5526.2002 PMC133943

[b16] Wang H. *et al.* Loss of granuphilin and loss of syntaxin-1A cause differential effects on insulin granule docking and fusion. J Biol Chem 286, 32244–32250 (2011). 21768089 10.1074/jbc.M111.268631 PMC3173164

[b17] Südhof T. C. & Rothman J. E. Membrane fusion: grappling with SNARE and SM proteins. Science 323, 474–477 (2009). 19164740 10.1126/science.1161748 PMC3736821

[b18] Izumi T., Kasai K. & Gomi H. Secretory vesicle docking to the plasma membrane: molecular mechanism and functional significance. Diabetes Obes Metab 9, 109–117 (2007). 17919185 10.1111/j.1463-1326.2007.00789.x

[b19] Izumi T. Heterogeneous modes of insulin granule exocytosis: molecular determinants. Front Biosci 16, 360–367 (2011). 10.2741/3692 21196175

[b20] Miyazaki J. *et al.* Establishment of a pancreatic β cell line that retains glucose-inducible insulin secretion: special reference to expression of glucose transporter isoforms. Endocrinology 127, 126–132 (1990). 2163307 10.1210/endo-127-1-126

[b21] Hanahan D. Heritable formation of pancreatic β-cell tumours in transgenic mice expressing recombinant insulin/simian virus 40 oncogenes. Nature 315, 115–122 (1985). 2986015 10.1038/315115a0

[b22] Efrat S. *et al.* Beta-cell lines derived from transgenic mice expressing a hybrid insulin gene-oncogene. Proc Natl Acad Sci USA 85, 9037–9041 (1988). 2848253 10.1073/pnas.85.23.9037 PMC282658

[b23] Radvanyi F., Christgau S., Baekkeskov S., Jolicoeur C. & Hanahan D. Pancreatic β cells cultured from individual preneoplastic foci in a multistage tumorigenesis pathway: a potentially general technique for isolating physiologically representative cell lines. Mol Cell Biol 13, 4223–4232 (1993). 8391634 10.1128/mcb.13.7.4223-4232.1993 PMC359972

[b24] Ohara-Imaizumi M., Nakamichi Y., Tanaka T., Ishida H. & Nagamatsu S. Imaging exocytosis of single insulin secretory granules with evanescent wave microscopy: distinct behavior of granule motion in biphasic insulin release. J Biol Chem 277, 3805–3808 (2002). 11751926 10.1074/jbc.C100712200

[b25] Degtyar V. E., Allersma M. W., Axelrod D. & Holz R. W. Increased motion and travel, rather than stable docking, characterize the last moments before secretory granule fusion. Proc Natl Acad Sci USA 104, 15929–15934 (2007). 17893335 10.1073/pnas.0705406104 PMC2000388

[b26] Ester M., Kriegel H.-P., Sander J. & Xu X. A density-based algorithm for discovering clusters in large spatial databases with noise in Proceedings of the Second International Conference on Knowledge Discovery and Data Mining (KDD-96) (eds Simoudis E., Han J. & Fayyad U.) 226–231 (AAAI Press, 1996).

[b27] Pertsinidis A. *et al.* Ultrahigh-resolution imaging reveals formation of neuronal SNARE/Munc18 complexes *in situ*. Proc Natl Acad Sci USA 110, E2812–2820 (2013). 23821748 10.1073/pnas.1310654110 PMC3725074

[b28] Nan X. *et al.* Single-molecule superresolution imaging allows quantitative analysis of RAF multimer formation and signaling. Proc Natl Acad Sci U S A 110, 18519–18524 (2013). 24158481 10.1073/pnas.1318188110 PMC3831949

[b29] Dudok B. *et al.* Cell-specific STORM super-resolution imaging reveals nanoscale organization of cannabinoid signaling. Nat Neurosci 18, 75–86 (2015). 25485758 10.1038/nn.3892 PMC4281300

[b30] Dempsey G. T., Vaughan J. C., Chen K. H., Bates M. & Zhuang X. Evaluation of fluorophores for optimal performance in localization-based super-resolution imaging. Nat Methods 8, 1027–1036 (2012). 22056676 10.1038/nmeth.1768 PMC3272503

[b31] Izumi T., Gomi H., Kasai K., Mizutani S. & Torii S. The roles of Rab27 and its effectors in the regulated secretory pathways. Cell Struct Funct 28, 465–474 (2003). 14745138 10.1247/csf.28.465

[b32] Kotake K. *et al.* Noc2, a putative zinc finger protein involved in exocytosis in endocrine cells. J Biol Chem 272, 29407–29410 (1997). 9367993 10.1074/jbc.272.47.29407

[b33] Waselle L. *et al.* Involvement of the Rab27 binding protein Slac2c/MyRIP in insulin exocytosis. Mol Biol Cell 14, 4103–4113 (2003). 14517322 10.1091/mbc.E03-01-0022 PMC207003

[b34] Mizuno K., Ramalho J. S. & Izumi T. Exophilin8 transiently clusters insulin granules at the actin-rich cell cortex prior to exocytosis. Mol Biol Cell 22, 1716–1726 (2011). 21441305 10.1091/mbc.E10-05-0404 PMC3093323

[b35] Wang H. *et al.* The Rab27a effector exophilin7 promotes fusion of secretory granules that have not been docked to the plasma membrane. Mol Biol Cell 24, 319–330 (2013). 23223571 10.1091/mbc.E12-04-0265 PMC3564536

[b36] Grosshans B. L., Ortiz D. & Novick P. Rabs and their effectors: achieving specificity in membrane traffic. Proc Natl Acad Sci U S A 103, 11821–11827 (2006). 16882731 10.1073/pnas.0601617103 PMC1567661

[b37] Takahashi N. *et al.* Two-photon fluorescence lifetime imaging of primed SNARE complexes in presynaptic terminals and β cells. Nat Commun 6, 8531 (2015). 26439845 10.1038/ncomms9531 PMC4600761

[b38] Sieber J. J. *et al.* Anatomy and dynamics of a supramolecular membrane protein cluster. Science 317, 1072–1076 (2007). 17717182 10.1126/science.1141727

[b39] Bar-On D. *et al.* Super-resolution imaging reveals the internal architecture of nano-sized syntaxin clusters. J Biol Chem 287, 27158–27167 (2012). 22700970 10.1074/jbc.M112.353250 PMC3411058

[b40] Tsuboi T. & Fukuda M. The Slp4-a linker domain controls exocytosis through interaction with Munc18-1.syntaxin-1a complex. Mol Biol Cell 17, 2101–2112 (2006). 16481396 10.1091/mbc.E05-11-1047 PMC1446092

