# Supplementary material for: Granuphilin exclusively mediates functional granule docking to the plasma membrane
Source: Sci Rep. 2016 Apr 1;6:23909. doi: 10.1038/srep23909 (PMC4817151; doi:10.1038/srep23909)
Supplement: Supplementary Information [file srep23909-s1.doc]

**Supplementary information**

**Granuphilin exclusively mediates functional granule docking to the plasma membrane**

Kouichi Mizuno^1^, Takuji Fujita^1^, Hiroshi Gomi^1^, and Tetsuro Izumi^1,2^*

^1^Laboratory of Molecular Endocrinology and Metabolism, Department of Molecular Medicine, Institute for Molecular and Cellular Regulation, Gunma University, ^2^Research Program for Signal Transduction, Division of Endocrinology, Metabolism and Signal Research, Gunma University Initiative for Advanced Research, Maebashi, Gunma 371-8512, Japan

*To whom correspondence should be addressed: Tetsuro Izumi, Department of Molecular Medicine, Institute for Molecular and Cellular Regulation, Gunma University, Maebashi, Gunma 371-8512, Japan. Tel.: 81-27-220-8856; Fax: 81-27-220-8860; E-mail: tizumi@gunma-u.ac.jp

**Supplementary Figure**

**
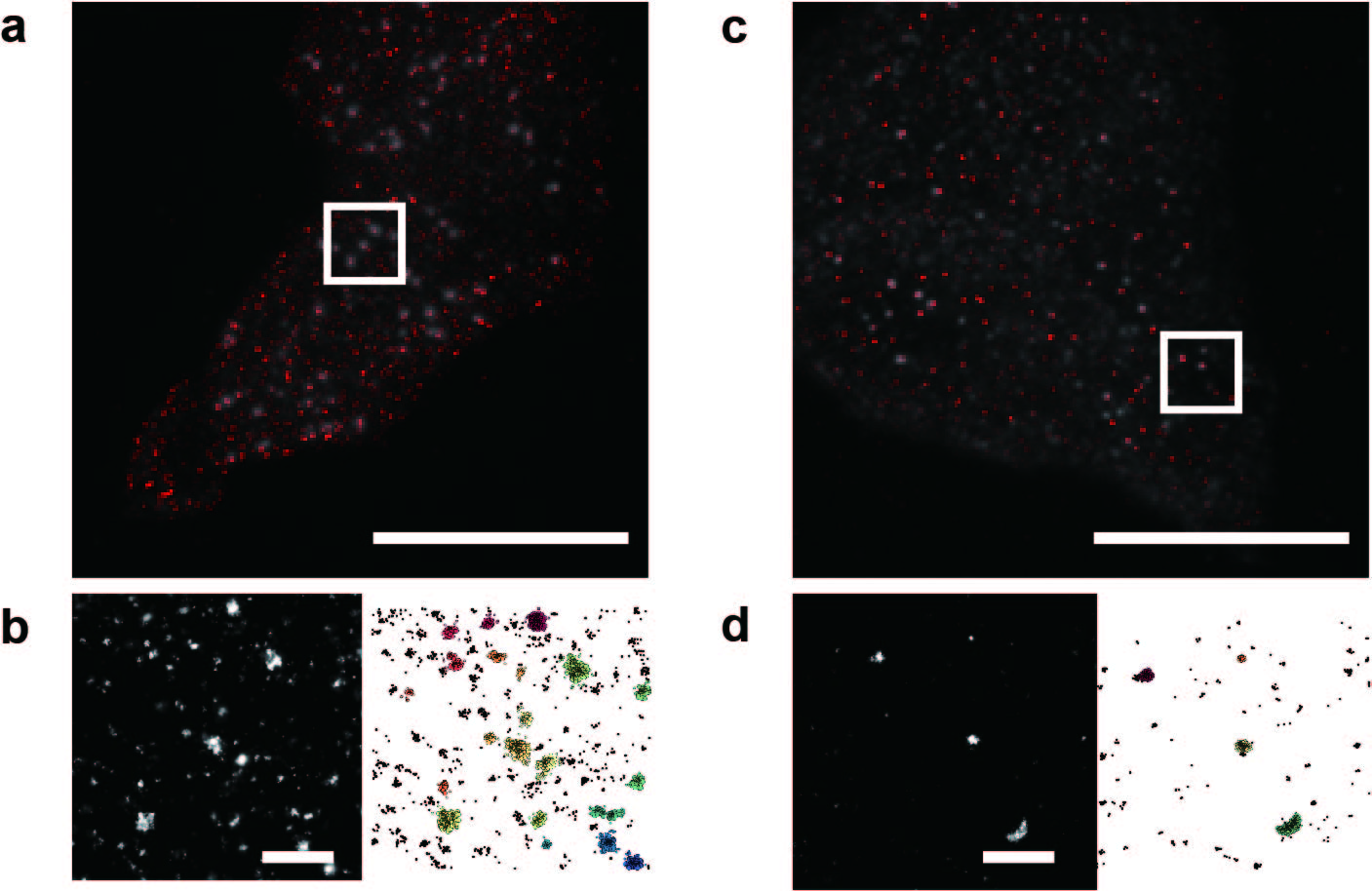
**

**Figure S1: Rab27a and Munc18-1 clusters in MIN6 cells.** MIN6 cells were coimmunostained with anti-granuphilin and either anti-Rab27a or anti-Munc18-1 antibodies. (**a**, **c**) Merged images of endogenous granuphilin by TIRFM (gray) and endogenous Rab27a (**a**) or Munc18-1 (**c**) by dSTORM (red). Bars, 5 μm. (**b**, **d**) dSTORM images (left) and their clusters identified by DBSCAN (right) of Rab27a (**b**) and Munc18-1 (**d**), from boxed regions in **a** and **c**, respectively. Bars, 500 nm.

**Supplementary video legends**

**Video S1: Granule mobility under TIRFM in a resting state of granuphilin-null β cells expressing Insulin-V and KuO-Grph.** The cells were observed for 10 s by TIRFM. The images were taken every 103 msec. Merged images of Insulin-V (green) and KuO-Grph (red) are shown. Note that granuphilin-positive granules exhibit severely restricted mobility, compared with granuphilin-negative granules. Bar, 10 μm.

**Video S2: Granule mobility under TIRFM in a resting state of granuphilin-null β cells** **expressing Insulin-V but not KuO-Grph.** The cells were observed for 10 s by TIRFM. The images were taken every 103 msec. Note that granules beneath the plasma membrane are mobile. Bar, 10 μm.

**Video S3: Depolarization-induced fusion of granuphilin-positive and -negative insulin granules in granuphilin-null β cells expressing Insulin-V and KuO-Grph.** The cells were stimulated by 60 mM KCl at time 0 s and were observed for 90 s by TIRFM. The images were taken every 103 ms. Insulin-V (left), KuO-Grph (middle), and merged images (right) are shown. Bar, 10 μm.

**Video S4: An example of the *passenger* type of exocytosis.**

Granuphilin-null β cells expressing Insulin-V and KuO-Grph were stimulated by 60 mM KCl under TIRFM. The images were taken every 103 ms. Insulin-V (left), KuO-Grph (middle), and merged images (right) are shown. Note that a granule without granuphilin fluorescence abruptly appears and fuse at time 2 s. Bar, 1 μm.

**Video S5: Behaviors of insulin granules and granule-associated granuphilin during exocytosis.** Granuphilin-null β cells expressing Insulin-V and KuO-Grph were stimulated by 60 mM KCl under TIRFM. The images were taken every 103 ms. Insulin-V (left), KuO-Grph (middle), and merged images (right) are shown. Note that the motion of granuphilin-positive granules destined to fuse at time 2 s increases just before exocytosis and that the fluorescence intensity of granule-associated granuphilin decreases during exocytosis. Bar, 1 μm.
